# *Bacopa monnieri* Promotes Neuronal Development by Regulating the Neurotrophin Signaling Pathway

**DOI:** 10.3390/ijms27073048

**Published:** 2026-03-27

**Authors:** Raju Dash, Sarmistha Mitra, Nayan Dash, Largess Barua, Kishor Mazumder, Il Soo Moon

**Affiliations:** 1 Department of Anatomy, College of Medicine, Dongguk University, Gyeongju 38066, Republic of Korea; dash_raju@dgist.ac.kr; 2 Department of New Biology, Daegu Gyeongbuk Institute of Science and Technology, Daegu 42988, Republic of Korea; 3 InnoCORE AI-CRED Institute, Korea Advanced Institute of Science and Technology (KAIST), Daejeon 34141, Republic of Korea; 4 GIST InnoCORE AI-Nano Convergence Institute for Early Detection of Neurodegenerative Diseases, Gwangju Institute of Science and Technology, Gwangju 61005, Republic of Korea; 5 Department of Integrative Biotechnology, College of Biotechnology and Bioengineering, Sungkyunkwan University, Suwon 16419, Republic of Korea; dashnayan.py@gmail.com; 6 Department of Anatomy and Neurobiology, School of Dentistry, Kyungpook National University, Daegu 41940, Republic of Korea; largessvishal@gmail.com; 7 Department of Pharmacy, Jashore University of Science and Technology, Jashore 7408, Bangladesh; kmazumder@just.edu.bd

**Keywords:** network pharmacology, *Bacopa Monnieri*, neuronal development, neuritogenesis, synaptogenesis

## Abstract

*Bacopa monnieri* (L.) Wettst. (Family: Scrophulariaceae) is a well-known edible plant used in ethnic and Ayurveda medicine for centuries to improve memory deficit, enhance cognitive function, and treat nervous system disorders. Despite accumulating in vivo evidence for its cognitive benefits, the detailed mechanisms by which its bioactive compounds act on primary neurons remain elusive. In the present study, we dissect the mechanism by which *Bacopa monnieri* promotes neuronal development by treating primary hippocampal neuronal cultures with its ethanolic extract (BMEE) and integrating insights from in silico network pharmacology. We identified that BMEE at different concentrations promotes neuritogenesis and has a remarkable impact on early neuronal maturation, and axonal and dendritic outgrowth. Also, BMEE regulated synaptic plasticity by increasing the expression of NMDA receptors. Metabolites of BMEE were identified by gas chromatography–mass spectrometry (GC-MS) analysis, from which a network pharmacology model was constructed, in which BMEE metabolites were projected to regulate the neurotrophin signaling pathway. Indeed, the BMEE-mediated neuritogenic effect was abolished by the presence of a TrkA receptor-specific inhibitor, suggesting that the neuritogenic effect of BMEE is TrkA-dependent. Also, molecular docking following MD simulations supported the idea that BMEE metabolites, particularly δ-Tocopherol and O-methyl-, bind with high affinity to the TrkA receptor (NGF-binding domain). This study collectively illuminates the TrkA-mediated pathway through which *Bacopa monnieri* promotes neuronal development and suggests that bioactive metabolites from BMEE might hold potential as a source for designing therapeutic agents for various cognitive disorders.

## 1. Introduction

Dementia, the decline in sensory perception and motor coordination, and neurodegeneration are common characteristics of brain aging [1] and are some of the most alarming phenomena in older people [2,3]. Neurodegeneration and other pathological features, like misfolded protein aggregates [4], aberrant neural network activity, neuronal death [5], and loss of synaptic plasticity [6], are all directly linked with different neurodegenerative, cognitive, and neuropsychiatric diseases like Alzheimer’s, Parkinson’s, Huntington’s, and amyotrophic lateral sclerosis. Unfortunately, there is no therapeutic strategy to cure these disorders. At the same time, there are some available pharmacological approaches (Cholinomimetics, anti-psychotic, NMDA receptor antagonist, and sedatives) that can improve the symptoms and help in the management of disease progression; however, these therapeutics may cause some unwanted side-effects, like hyperlipidemia, urinary tract infection, diabetes, diarrhea, constipation, nausea, sleep disorder, hypertension, bradycardia, and so on [7,8]. Intriguingly, it is possible to improve the symptoms of neurodegeneration by introducing various neurotropic factors and neurotrophins to promote neuronal differentiation and stimulate synaptic connections [9,10].

Nevertheless, due to challenges in protein transport into the brain and poor pharmacokinetic properties, neurotrophins and neurotrophic factors still fail to demonstrate a clear therapeutic benefit [11,12]. In such conditions, small molecules that bind to neurotrophin receptors and activate or enhance their activity may offer promising clinical benefits for the treatment of neurodegenerative diseases. Surprisingly, phytochemicals present in many plants, herbs, and natural products have been reported in recent years that can modulate neuronal growth, enhance neuronal differentiation, promote synapse formation by working on multiple different neuronal pathways, and can be easily introduced as an alternative therapy besides other therapeutics [13,14] because they are cost-effective, cause minimal or no side effects, and are readily available [15].

The main focus of this study is to investigate the impact and possible mechanistic pathway of a traditional ayurvedic plant of Southeast Asian origin, *Bacopa monnieri* (L.) Wettst. (Family: Scrophulariaceae), on neuronal development. *Bacopa monnieri* is a well-known herb for its neuropharmacological and neurotropic effects [16,17] and its beneficial role in treating neurodegenerative diseases [18]. Notably, *Bacopa monnieri* is used therapeutically and is edible, making it a convenient dietary supplement for promoting brain health. In Ayurveda, Bacopa is believed to improve cognition, reduce anxiety, modulate intellect, and recover concentration [19,20,21]. Several in vivo studies also reported the neuroprotective effect of the Bacopa plant on animal models of Parkinson’s disease [22,23,24], Alzheimer’s disease [25,26,27], and dementia [28,29]. Similarly, it has been reported to improve cognitive function, promote learning ability, and memory [30,31] and improve the level of neurotransmitters [32]. Other studies showed that *Bacopa monnieri* extract administration in different in vivo models increases neurotrophin levels such as NGF and BDNF [33,34] and regulates glutamatergic systems in response to neuronal protection [32,33,35]. However, the mechanism by which *Bacopa monnieri* extract regulates neurotrophin expression and glutamatergic systems remains unclear, and it is unclear whether *Bacopa monnieri* extract can promote neuronal development and differentiation, especially in primary neurons.

**Figure 1:**
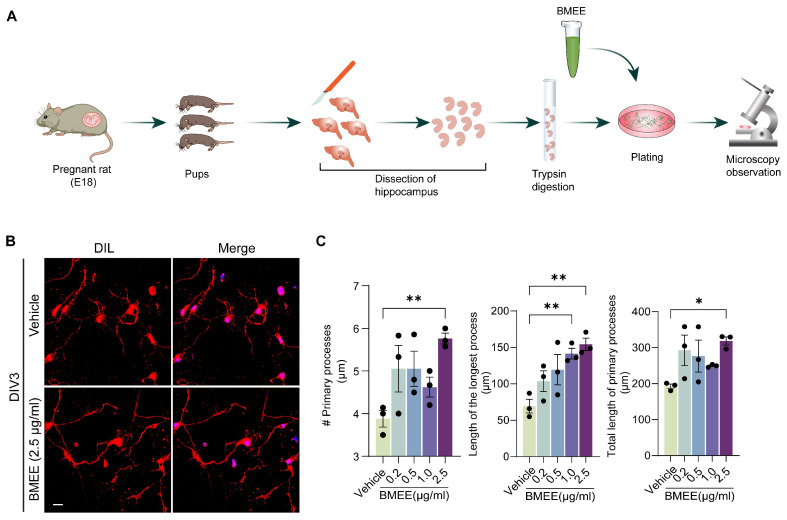
Primary cultured neurons showed significant neurite outgrowth by application of an optimized dose of BMEE. (**A**) Pictorial representation of experimental procedure. Dissection of pregnant mice, collection of hippocampi of mouse pups, and plating of the neuronal cells. (**B**) Representative fluorescent images of Dil (Red) and DAPI (Blue) counter-stained primary hippocampal neurons grown until DIV3 of the culture in BMEE-treated (2.5 μg/μL) and untreated conditions. Scale bar, 20 μm. (**C**) Statistical analysis of morphometric parameters of neuronal development following different doses of BMEE treatment, including the number of primary neuronal processes, the length of the longest process, and the total length of all primary processes. Data are presented as mean ± SEM from three independent experiments (*n* = 3), with 10 neurons analyzed per experiment. Statistical significance was determined by one-way ANOVA followed by Tukey’s post hoc test, with comparisons made against the vehicle control (* *p <* 0.05, ** *p <* 0.01). Here, # indicates the “number of”.

In our present work, the crude ethanolic extract of the whole plant of *Bacopa monnieri* (BMEE) was examined in primary cultured hippocampal neurons (Figure 1A) using a combination of in silico and in vitro approaches. As the study is designed to explore the mechanistic pathway of BMEE, we conducted gas chromatography–mass spectroscopy (GC-MS) analysis to detect the active metabolites present in the extract. Based on the identified metabolites, network pharmacology analysis is also used to identify targets associated with the active components and their potential regulated signaling pathways. Guided by the network pharmacology approach, our study reported that the metabolites present in BMEE regulate signaling pathways of axonal and dendritic development and early neuronal maturation in a TrkA receptor (*NTRK1*)-dependent manner.

## 2. Results

### 2.1. Neuronal Growth Promoted by BMEE

To determine whether BMEE can accelerate neuronal growth, we first examined BMEE concentrations (0.1–2.5 µg/mL) in a primary neuronal culture derived from rat hippocampus and observed changes in growth parameters by DIV3.

Investigation of the basic neurite growth parameters (length of the longest process, total number of primary processes, and sum of all the processes) from the phase-contrast images showed that BMEE substantially promoted morphological development of neurons compared to the control vehicle group (Figure 1). Among the concentrations tested, 2.5 μg/mL BMEE was the most effective in enhancing neuronal growth parameters, including the number of primary processes, compared with the vehicle control (Figure 1B,C). At the same concentration, BMEE also significantly increased the lengths of both the primary and the longest processes by approximately 1.6-fold and 3-fold, respectively, relative to the vehicle group (Figure 1C). In addition, BMEE concentrations up to 2.5 μg/mL did not produce detectable cytotoxicity in neurons, as assessed by the trypan blue exclusion assay at DIV8, in which live and dead cells were counted (Figure S1, Supplementary File S2). Together, these results indicate that BMEE promotes neuronal growth under the present experimental conditions. Higher concentrations were not further evaluated in the viability assay because preliminary observations indicated restricted neuronal growth during earlier stages of culture.

In addition, the neuritogenic effect of BMEE was comparable with that of a known standard neuro-modulator, stigmasterol [36]. At DIV3, the neuritogenic effect of BMEE parameters was superior to that of stigmasterol across all morphometric analysis parameters (Figure S2A,B, Supplementary File S2).

### 2.2. BMEE-Mediated Neuronal Development Involves Multiple Signaling Pathways

Since 2.5 µg/mL of BMEE showed significant neuritogenic effects in primary neuronal culture, we next performed phytochemical screening by GC-MS to identify the active compounds present in BMEE (Table S1, Supplementary File S2). A total of 24 compounds were detected by GC-MS analysis, and some were identified at two different retention times (RTs) with different peak areas, which may be due to isomeric species, related derivatives, or overlapping signals in the complex extract. The names of the compounds, their retention time, their peak area (%), and their structures are illustrated in Table S1 (Supplementary File S2). The percentage of peak area indicates that two metabolites, δ-Tocopherol, O-methyl- and 9-Octadecenamide, (Z)-, were abundant in BMEE.

**Figure 2:**
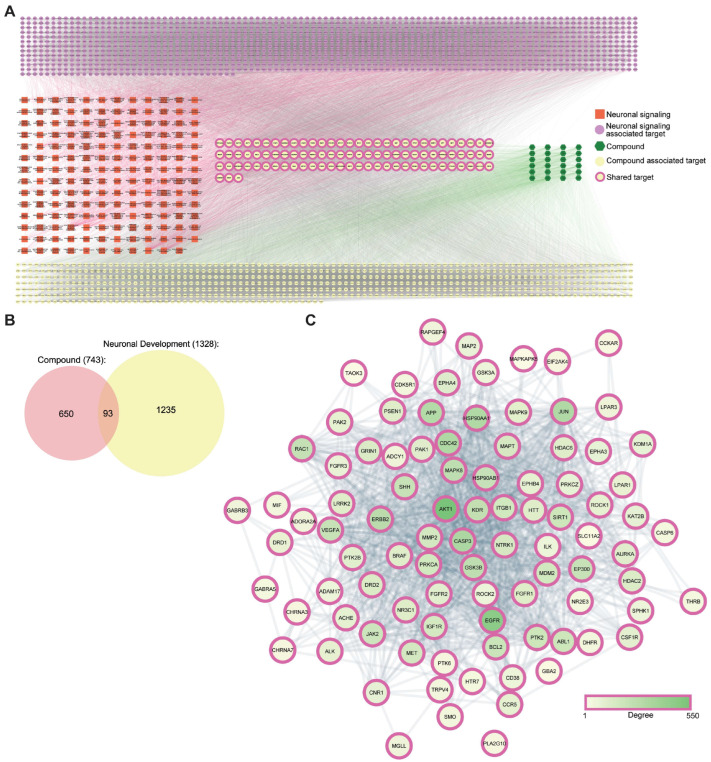
BMEE is involved in neuronal development signaling pathways. (**A**) Mechanistic analysis from network pharmacology represents active BMEE metabolisms in neuronal signaling networks. Here, green hexagon-shaped nodes represent metabolites identified in BMEE by GC-MS (Table S2, Supplementary File S2) whereas red square boxes indicate the neuronal developmental signaling pathways involved. The purple circular nodes illustrate the nuclear signaling-associated targets, and the yellow circular nodes represent the targets known to associate with metabolites. Also, the yellow circular nodes with purple border lines represent shared targets. (**B**) Venn diagram showing the common targets between active compounds BMEE and neuronal development. (**C**) The protein–protein interaction (PPI) network represents the interconnectedness between 93 targets of BMEE, which also belong to neuronal developmental signaling. In this representation, circular nodes represent targets, and blue lines represent their interactions. The darkness of the node colors represents the degree of connectivity.

**Figure 3:**
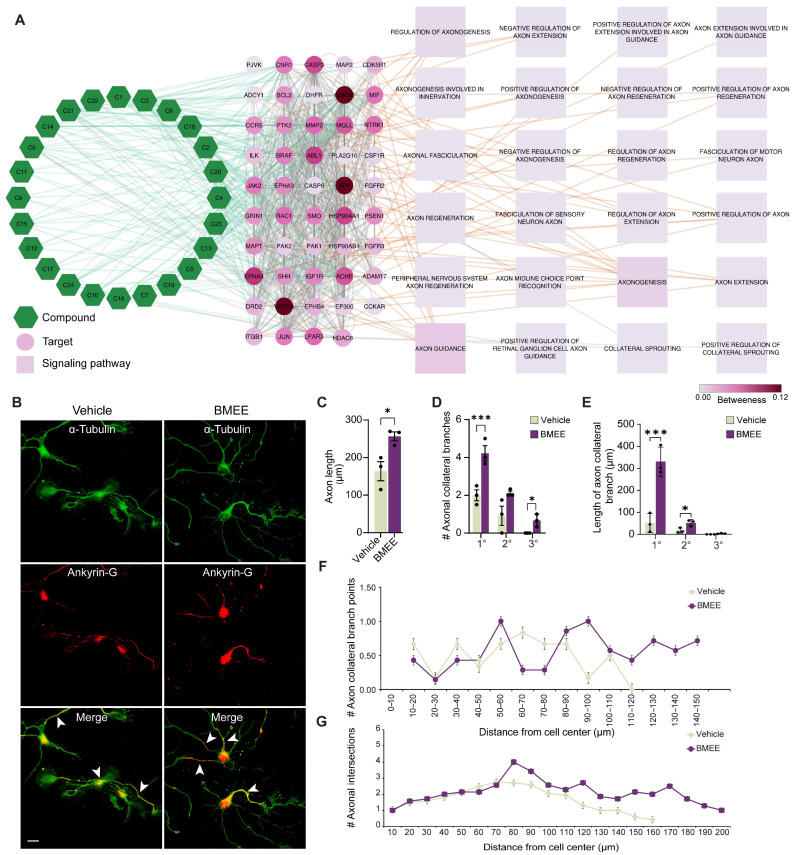
Axonal growth parameters are promoted by BMEE. (**A**) Network construct illustration showing BMEE’s involvement in axonal growth pathways. The green hexagons denote the compounds, the circular nodes denote the targets, and the square boxes represent the signaling pathways. The lines represent the interconnection among compound–target–signaling pathways. The degree of connectivity is indicated by the darkness of the target and signaling pathway symbols. (**B**) Fluorescent images of neurons immunostained with Ankyrin–G (red, axonal marker) and α–Tubulin (green, cytoskeleton marker) antibody. Here, primary hippocampal neurons were grown until DIV 5, fixed, and immunostained as described in Figure 1, in the presence of vehicle and/or BMEE (2.5 μg/mL). Cell morphology is visualized by tubulin, and white arrows mark axons. Scale bar = 20 μm for all images. Morphometric analysis of axonal development in BMEE–and vehicle-treated neurons showing (**C**) axon length, (**D**) number of axon collateral branches at different branching orders (primary, secondary, or tertiary), and (**E**) length of axon collateral branches at different branching orders (primary, secondary, or tertiary). Sholl analysis is shown in (**F**) for the number of axon collateral branching points and in (**G**) for axonal intersection points. Data are expressed as mean ± SEM from three independent experiments (*n* = 3), with 5–10 neurons analyzed per experiment. Statistical significance was determined using a two-tailed Student’s *t*-test (* *p <* 0.05, *** *p <* 0.001). In (**F**,**G**), # denotes the “number of”.

**Figure 4:**
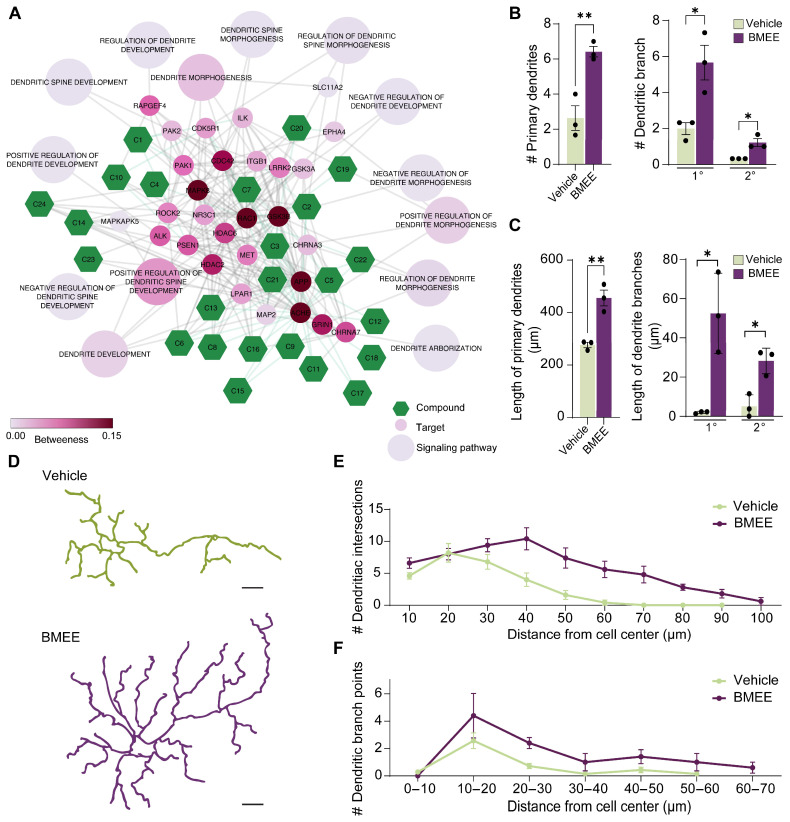
BMEE components accelerate dendritic arborization. (**A**) Various dendritic developmental pathways are modulated by BMEE-derived active components, as revealed by network pharmacology screening. The green hexagons denote compounds, and the small and large circular nodes represent targets and signaling pathways, respectively. The connecting lines show interconnections. Statistical representation of dendritic morphogenesis in vehicle- and BMEE-treated neurons, including (**B**) number of primary dendrites at different branching orders (primary or secondary) and number of dendritic branches, (**C**) length of primary dendrites, and length of dendritic branches. Representative Sholl analysis of vehicle- and BMEE-treated neurons at DIV5 is shown in (**D**). Scale bar = 20 μm. Statistical evaluation of Sholl analysis is presented in (**E**) number of dendritic intersections and (**F**) dendritic branching points. Data are presented as mean ± SEM from three independent experiments (*n* = 3), with 5–10 neurons analyzed per experiment. Statistical significance was determined using a two-tailed Student’s *t*-test (* *p <* 0.05, ** *p <* 0.01). In (**B**,**E**,**F**), # indicates the “number of”.

**Figure 5:**
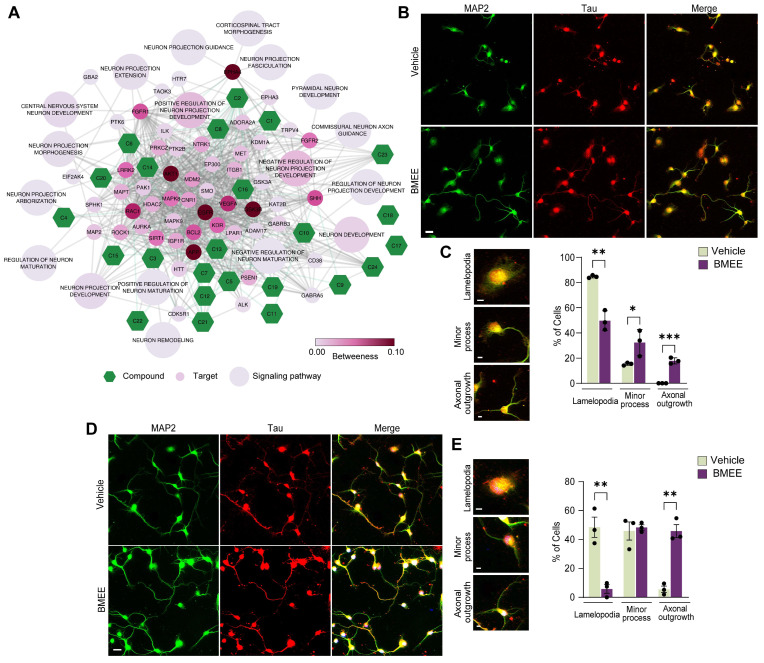
Neuronal development in the early stages can be accelerated by BMEE. (**A**) Compound–target–neuronal development signaling pathway network. The green hexagons denote compounds, and the small and large circular nodes represent targets and signaling pathways, respectively. (**B**) Fluorescent images showing neurons grown until 24 h in the same culture condition with vehicle/BMEE (2.5 μg/mL), fixed and immunostained with MAP2 (Green, neuronal marker) and Tau (Red, dendritic marker) antibody. (**C**) Different developmental stages were observed at 24 h: lamellipodia, minor processes, and axonal outgrowth (insets). Statistical analysis shows the percentage of neurons in different stages at 24 h in vehicle- or BMEE-treated conditions. (**D**) Fluorescent images showing neurons grown until 48 h in the same culture condition with vehicle/BMEE (2.5 μg/mL), fixed and immunostained with MAP2 and Tau antibody. (**E**) Different developmental stages were observed at 48 h: lamellipodia, minor processes, and axonal outgrowth (insets). Statistical analysis shows the percentage of neurons in different stages at 48 h in vehicle- or BMEE-treated conditions. Scale bar, 20 μm for all image panels (**B**,**D**) and 2 μm for panels (**C**,**E**). Statistical significance was determined using a two-tailed Student’s *t*-test. Data are presented as mean ± SEM from three independent experiments (*n* = 3), with 100–150 neurons analyzed in each experiment (* *p <* 0.05, ** *p <* 0.01, *** *p <* 0.001).

Next, we identified potential targets for these 24 compounds using in silico target-fishing methods described in the method section. In in silico target prediction, 743 targets were unveiled by 24 compounds after the duplicates were removed (Figure 2A and Supplementary File S1). In addition, 1328 targets were associated with neuronal developmental pathways from the GO database. A Venn diagram was constructed to identify common targets between compounds and neuronal signaling (Figure 2B), revealing 93 targets associated with neuronal signaling genes. Considering these 93 shared targets, a protein–protein interaction (PPI) network was constructed (Figure 2C). The PPI network showed higher connectivity among some targets, suggesting several significant targets associated with neuronal development. Among the potential targets, Akt1, EGFR, HSP90AA1, BCL2, CASP3, JUN, HSP90AB1, ERBB2, and GSK3B showed high degrees of connectivity. Furthermore, several important signaling cascades of neuronal development were found to associate with these targets, especially those involved in axon (Figure 3A), dendrite development (Figure 4A), and neuronal differentiation (Figure 5A).

### 2.3. BMEE Regulates Different Stages of Neural Development

#### 2.3.1. BMEE Influences Axonal Development and Branching

Since network pharmacology revealed that BMEE components are interconnected with different axonal pathways, including axonogenesis, axon guidance, and positive regulation of axon extension, they showed high betweenness centrality in the network (Figure 3A). Therefore, we further assessed axonal development by BMEE in primary cultured neurons.

Morphological analysis of DIV5 neurons showed increased axonal length when treated with BMEE. The axons can be easily distinguished from the dendrites by double immunostaining for tubulin and ankyrin G (Figure 3B). Figure 3C shows that axonal length was 76.8% longer in neurons treated with BMEE compared to neurons treated with vehicle, and that treatment also significantly increased the quantity and length of the primary and secondary axonal collateral branches (Figure 3D,E).

In order to study axonal sprouting and branching locations, Sholl analysis was applied to DIV5 neurons. The number of collateral branching points was 1.5 times higher in the BMEE compared to the vehicle (Figure 3F), meaning that BMEE-treated neurons could be observed up to 150 μm beyond the Sholl circle. However, control neurons did not exhibit any collateral branching points beyond 120 μm. Again, a 1.59-fold improvement in the axonal crossing of the Sholl concentric circle was revealed in the case of BMEE-treated neurons (Figure 3G). In addition, axonal crossings along the Sholl concentric circles were found to be up to 200 μm in the investigation, whereas control neurons only showed crossings up to 160 μm. The results show that BMEE significantly affects sprouting and axonal maturation.

#### 2.3.2. BMEE Promotes Dendritic Architecture

Among the different dendritic development signaling pathways, dendrite development, positive regulation of dendrite morphogenesis, and positive regulation of dendritic spine development are found to be most influential in the network of BMEE metabolites (Figure 4A). Therefore, the effect of BMEE on dendrite arborization via morphological analysis on DIV5 neurons was further incorporated.

The morphological analysis revealed that BMEE increased dendrite length by around 40% compared to the vehicle-treated groups (Figure 4C). Likewise, BMEE treatment significantly elevated the number and length of primary and secondary branching of dendrites as well (Figure 4B,C). Again, the Sholl profile investigated dendritic intersections and branching points, which showed increases of 2.21-fold (Figure 4E) and 2.75-fold (Figure 4F), respectively, compared with vehicle-treated neurons. The line drawings of neurons shown in Figure 4D illustrate greater dendritic branching in BMEE-treated neurons.

#### 2.3.3. BMEE Modulates Early Neuronal Differentiation

As the in silico analysis suggested an association between BMEE metabolites and the neuronal development and maturation signaling pathway (Figure 5A), we performed further analysis in cultured neurons. The best-optimized dose of BMEE was tested for its effect on early neuronal maturation. The number of neurons at 24 h and 48 h of neuronal culture was counted. Neuronal maturation is classified into three classes: lamellipodia (stage 1), minor process (stage 2), and axonal elongation (stage 3). Immunofluorescence images of neurons fixed at 24 h and 48 h, stained with a somatodendritic marker (MAP2) and axonal marker (Tau), are represented in Figure 5B and Figure 5D, respectively. Also, different early growth stages of neurons are shown in insets (Figure 5C,E).

Cultures treated with BMEE showed approximately 40% and 20% of cells in developmental stages 2 and 3, respectively, at 24 h (DIV1). In contrast, around 20% of vehicle-treated neurons reached stage 2. Furthermore, no neurons reached the 3rd stage of maturation (Figure 5C). At 48 h, the elongation of axons in BMEE-treated neurons at stage 3 was around 5 times more than the vehicle-treated neurons (Figure 5E). These findings suggest that BMEE stimulated neuronal differentiation.

### 2.4. Neuronal Development by BMEE Through the TrkA Signaling Pathway

#### 2.4.1. BMEE Differentially Regulates Common Targets in Neuronal Development

**Figure 6:**
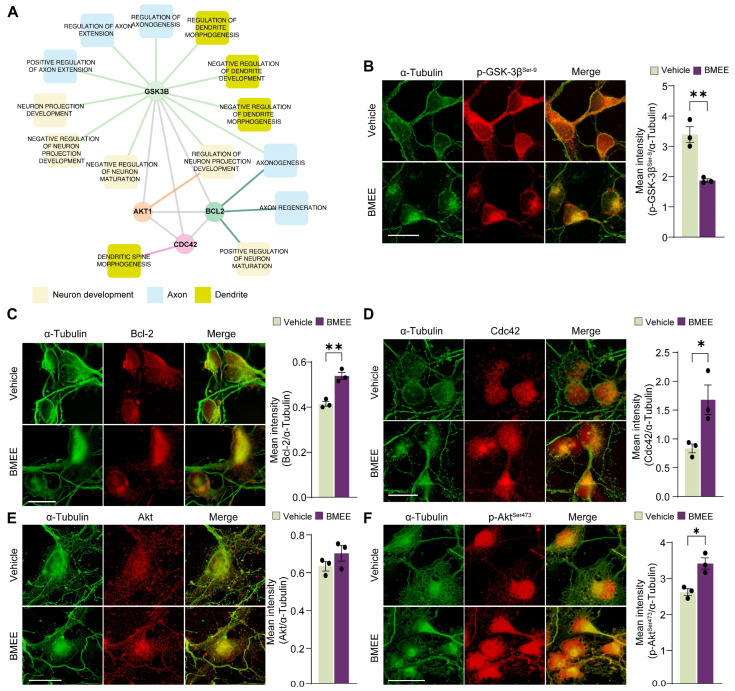
BMEE increases the expression of different genes of the neurotrophin signaling system. (**A**) Protein–protein interaction (PPI) network derived from GO enrichment analysis highlighting candidate regulators associated with neuronal development. GSK3β, Akt1, Bcl-2, and Cdc42 were identified as central nodes linked to biological processes involved in axonogenesis, dendrite morphogenesis, neuronal projection development, and neuronal maturation. Nodes represent proteins or biological processes, and connecting edges indicate functional associations. Color coding denotes neuronal developmental categories: neuron development (beige), axon-related processes (blue), and dendrite-related processes (bright green-yellow). Primary cultured neurons were grown until DIV3 in BMEE/vehicle, which was then fixed and double-stained with anti-α-tubulin (control) and antibodies for the indicated proteins. (**B**) Representative fluorescence image of α-tubulin (green) and phospho-GSK3β (red), and statistical quantification of relative mean fluorescence intensity (compared with α-tubulin) in either vehicle/BMEE. (**C**) Representative fluorescence image of tubulin (green) and Bcl-2 (red) and statistics. (**D**) Representative fluorescence image of tubulin (green) and Cdc42 (red) and statistics. (**E**) Representative fluorescence image of tubulin (green) and Akt (red), and statistics. (**F**) Representative fluorescence image of tubulin (green), phospho-Akt (red), and statistics. Statistical significance was determined using a two-tailed Student’s *t*-test (* *p <* 0.05, ** *p <* 0.01). Data are presented as mean ± SEM from three independent experiments (*n* = 3), with 10 neurons analyzed per experiment. Scale bar = 20 μm for all images.

To identify the common factors through which BMEE may regulate axonal growth, dendritic development, and early neuronal maturation, we further analyzed the GO enrichment and PPI network data, which identified GSK3β, Bcl-2, Akt1, and Cdc42 as candidate regulators (Figure 6A). Importantly, these factors were associated with biological pathways directly relevant to the cellular effects observed in our study, including neuritogenesis, axonogenesis, dendrite morphogenesis, neuronal projection development, and early neuronal differentiation. Because these molecules are known to play critical roles in early neurogenesis and neuronal development [37,38,39,40,41,42], we further evaluated the expression of these four factors in BMEE- and vehicle-treated neurons by immunocytochemistry followed by fluorescence intensity analysis. Quantitative analysis showed that BMEE treatment significantly increased the expression levels of Bcl-2 (Figure 6C) and Cdc42 (Figure 6D), whereas total Akt1 expression was not significantly altered (Figure 6E). However, BMEE significantly increased Akt1 phosphorylation (Figure 6F), while GSK3β phosphorylation was reduced (Figure 6B). Together, these findings indicate that BMEE differentially regulates key factors involved in neuronal growth.

#### 2.4.2. Neuronal Development by BMEE Is Dependent on TrkA Receptor (NTRK1) Activation

**Figure 7:**
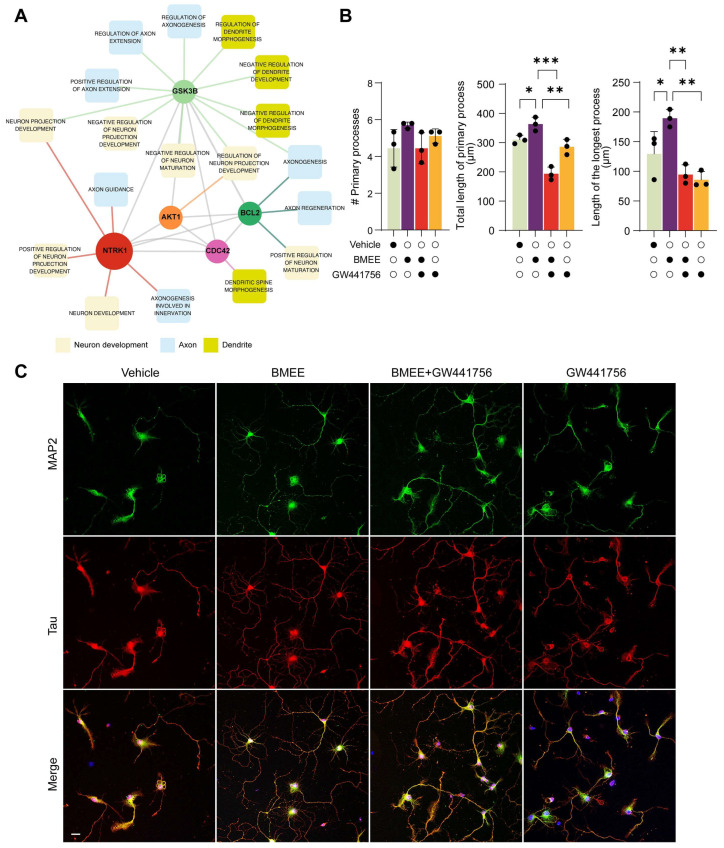
BMEE promotes neurite outgrowth by activating the TrkA pathway. (**A**) GO analysis illustrates the involvement of the TrkA receptor (*NTRK1*) with other targets and in neuron, dendrite, and axonal development. (**B**) Statistical analysis shows morphometric parameters of DIV 3 neurons: the number of primary processes, the total length of primary processes, and the length of the longest process in the presence of vehicle, BMEE (2.5 μg/mL), BMEE + GW441756 (TrkA inhibitor), and GW441756. Data are presented as mean ± SEM from three independent experiments (*n* = 3), with 10 neurons analyzed in each experiment. Statistical significance was determined by one-way ANOVA followed by Tukey’s post hoc test, with comparisons made against the vehicle control (* *p <* 0.05, ** *p <* 0.01, *** *p <* 0.001). (**C**) Representative fluorescent images show primary neurons grown to DIV 3, stained with MAP2 (Green) and Tau (Red), with DAPI in the vehicle/BMEE/BMEE + GW441756/GW441756-treated condition. “#” denotes “number of”. Scale bar, 20 μm, applies to all images.

Because GSK3β, Akt1, Bcl-2, and Cdc42 are well-established regulators of neuronal differentiation, neurite outgrowth, and cytoskeletal remodeling and are also associated with neurotrophin-related signaling pathways, we next investigated whether an upstream neurotrophin receptor might mediate the effects of BMEE. KEGG pathway enrichment and GO analyses indicated that TrkA (*NTRK1*), a key receptor in neurotrophin signaling, is connected to the regulation of these factors within the neurotrophin signaling pathway (Figure 7A; Figure S3, Supplementary File S2). Activation of TrkA is known to initiate multiple intracellular signaling cascades, including the PI3K/Akt pathway and small GTPase signaling [43], which regulate neuronal survival, cytoskeletal dynamics, and neurite outgrowth. Because BMEE treatment differentially regulated these four factors, we hypothesized that the neuronal growth-promoting effects of BMEE may be, at least in part, mediated through TrkA-dependent signaling.

Using morphometric analysis on DIV3 neurons, we further checked whether BMEE effects on neuronal growth can be blocked by the TrkA receptor inhibitor (GW441756). Interestingly, co-treatment with a TrkA blocker and BMEE significantly inhibits BMEE-induced neuronal growth. All the basic growth parameters of BMEE-treated DIV 3 neurons were found to reverse when the TrkA inhibitor was co-treated with BMEE (Figure 7B). Immunofluorescent images of neurons also showed that the TrkA inhibitor decreased neuronal growth compared to what was upregulated by BMEE treatment (Figure 7C). This analysis and its outcomes prove that TrkA is an essential and predominant pathway for BMEE-derived neuronal development.

#### 2.4.3. BMEE Positively Modulates Synaptogenesis

**Figure 8:**
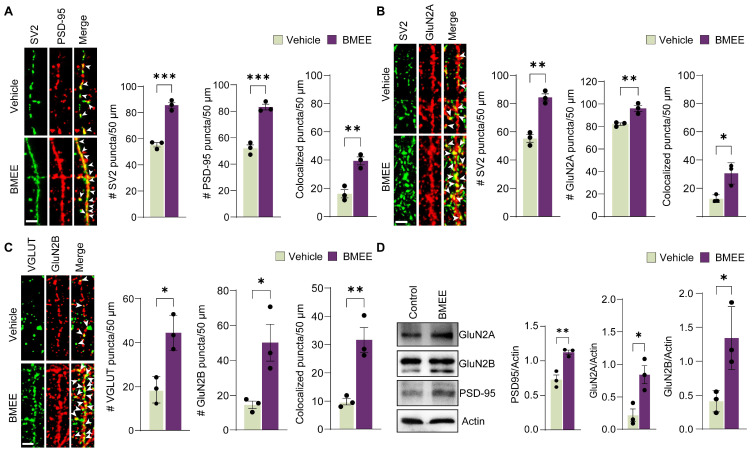
Synaptogenesis is promoted by BMEE. Cultured neurons were treated with BMEE (2.5 μg/mL) or vehicle and grown under the previously mentioned conditions until 16 days. After fixation, neurons were double-immunostained with pre- and postsynaptic markers. (**A**) Co-staining of a presynaptic marker, SV2 (green), and a postsynaptic marker, PSD-95 (red), represented in fluorescent images, and their co-localization (indicated by white arrows) shows synapse formation. Quantification analyses of puncta numbers along 50 μm dendrite length for SV2, PSD-95, and synapse. (**B**) Immunostained images showing individual synaptic puncta for SV2, GluN2A, and SV2/GluN2A colocalized puncta present a synapse (indicated by arrows) and quantification analysis of immunofluorescence staining puncta along a 50 μm length of dendrite; SV2, GluN2A, and synapse. (**C**) Immunostained images showing individual synaptic puncta for SV2 with GluN2B and SV2/GluN2B colocalized puncta present a synapse (indicated by arrows) and quantification analysis of immunofluorescence staining puncta along a 50 μm length of dendrite; SV2, GluN2B, and synapse. (**D**) Immunoblotting analysis of relative expression of PSD95, GluN2A, and GluN2B in DIV16 neurons, compared with actin. The first panel shows representative immunoblot analyses of GluN2A, GluN2B, PSD-95, and Actin, whereas the second panel highlights the mean intensity of the expressed proteins, measured using ImageJ (version 1.54) and expressed as % of control (average of triplicate). Scale bar, 2 μm, applies to all images. Statistical significance was determined using a two-tailed Student’s *t*-test (* *p <* 0.05, ** *p <* 0.01, *** *p <* 0.001). Data are presented as mean ± SEM from three independent experiments. For immunostaining, three independent experiments were performed (*n* = 3), with 10 neurons analyzed per experiment. Immunoblotting experiments were also repeated three times.

TrkA activation is also required for synaptogenesis [44], so we examined whether BMEE promotes synaptogenesis. The effect of BMEE on synaptogenesis was investigated in neurons grown on BMEE-containing media until DIV16. Then, neurons were fixed and immunostained with excitatory synaptic proteins, presynaptic (synaptic vesicle protein 2, SV2) and postsynaptic (postsynaptic density protein 95, PSD-95) markers. From fluorescent images, the density of both SV2 and PSD-95 puncta was calculated. It was revealed that BMEE significantly improved the density of both proteins (Figure 8A). Also, the density of SV2 and PSD-95 colocalized puncta was counted and found to be in higher numbers in the case of BMEE-treated groups (Figure 8A). Further validation was done by performing an immunoblotting assay. BMEE treatment upregulated PSD-95 expression (Figure 8D). To investigate the role of BMEE in inhibitory neurons, we used GAD6 and GABA_B_R1 as presynaptic and postsynaptic markers, respectively. BMEE significantly promoted the density of inhibitory synaptic marker proteins (Figure S4, Supplementary File S2). These findings suggest that BMEE promotes synaptogenesis.

Again, TrkA activation regulates N-methyl-d-aspartate (NMDA) receptor activity and synaptic plasticity [45]. Therefore, the expressions of N-methyl-d-aspartate (NMDA) receptor subunits GluN2A and GluN2B were analyzed for BMEE-treated neurons at DIV16. We considered only those NRs colocalized with presynaptic terminals, indicating synaptic ones. We used GluN2A and GluN2B antibodies, along with the presynaptic marker SV2 antibody, as shown in Figure 8B,C. The results showed that both NMDA subunit receptor expressions were significantly upregulated, and the number of SV2 and GluN2A colocalized puncta increased 1.45-fold in the BMEE-treated group. The statistical analysis, compared with the vehicle, shows that synapse density significantly increased, as seen in Figure 8B,C. We also performed immunoblotting to confirm the results (Figure 8D), which revealed that GluN2A and GluN2B band intensities were elevated. The Western blot and statistical analysis are shown in Figure 8D. The results presented here add to the growing evidence that BMEE modulates synaptic remodeling by enhancing both NMDA subunits.

### 2.5. Structure-Based Screening of BMEE Compounds for Potential TrkA Receptor Activator

**Figure 9:**
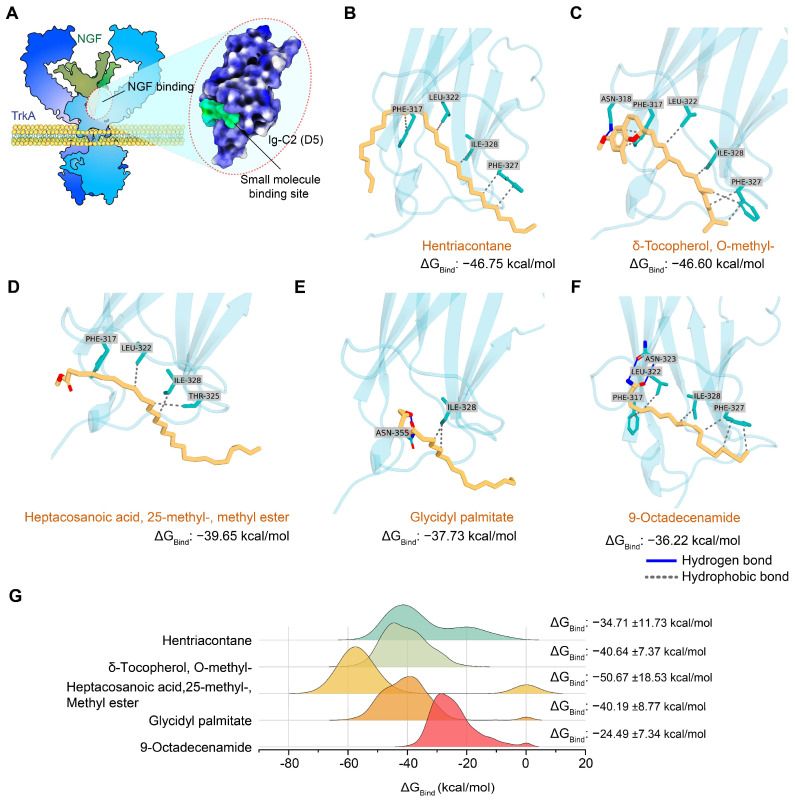
Structure–guided virtual screening reveals potential TrkA receptor activators from BMEE metabolites. (**A**) Using structural modeling and molecular dynamics (MD) simulations, prospective bioactive components interacting with the TrkA extracellular domain have been identified. The green dots represent binding sites for BDNF mimetic design, while the dotted circles provide a schematic of the BDNF–TrkA interaction. Ig stands for immunoglobulin. Molecular docking study of the interactions between the TrkA receptor and the top five hits, along with the MM–GBSA binding energies of these hits, such as for (**B**) Hentriacontane; (**C**) δ–Tocopherol, O–methyl–; (**D**) Heptacosanoic acid, 25–methyl–, methyl ester; (**E**) Glycidyl palmitate; and (**F**) 9–Octadecenamide, (z)-. In this figure, the gray dotted line shows the hydrophobic interaction, and the blue line indicates the hydrogen bond interaction. (**G**) Distribution and average binding energy of the top 5 hits calculated from the last 100 ns of MD–simulated trajectories, based on the MM–GBSA approach.

Again, TrkA receptor activation is upstream of the targets of BMEE and has been identified to play a substantial role in regulating BMEE-mediated neuronal development. We considered this receptor for molecular docking analysis to identify possible metabolites that bind to it (Figure 9A). Therefore, a virtual screening campaign comprising Glide XP docking and MM-GBSA binding energy analysis was performed to identify potential hits with high affinity for the TrkA receptor. Following molecular docking, MM-GBSA binding energy analysis showed that BMEE metabolites have an affinity for TrkA, with binding energies ranging from −46.75 to −14.29 kcal/mol (Table S3, Supplementary File S2). Five hits with the highest binding energy were identified, and detailed molecular interactions are visualized in Figure 9B–F. Docking-based intermolecular interaction analysis suggests that ILE328, PHE317, LEU322, and PHE327 residues of the TrkA receptor remain major contributors in the binding of these metabolites. However, whether these interactions were essential for binding energy has been further validated through molecular dynamics simulation and MM-GBSA analysis.

The top 5 hits from the molecular docking analysis were further subjected to MD simulation (Figure S5), and binding energies were calculated from simulation trajectories (Figure 9G). Analysis of binding energy based on MD simulation conformers suggests three metabolites, heptacosanoic acid, 25-methyl-, methyl ester; δ-Tocopherol, O-methyl-; and glycidyl palmitate, enduring high binding energy during simulation (Figure S5, Supplementary File S2). Although heptacosanoic acid, 25-methyl- showed high binding energy during the simulation, it did not form sustained intermolecular interactions with any specific receptor residue; instead, it formed minor interactions with many residues. Comparatively, SER304, PRO302, and LEU333 from the TrkA receptor remained major contributors to glycidyl palmitate binding (Figure S6A, Supplementary File S2), which was found absent in docking studies. On the other hand, residues such as PHE317, ASN318, LEU322, ILE328, and VAL354 were found to maintain stable interactions with δ-Tocopherol-O-methyl throughout the simulation (Figure S6B, Supplementary File S2), with more residue interactions consistent with molecular docking analysis.

## 3. Discussion

**Figure 10:**
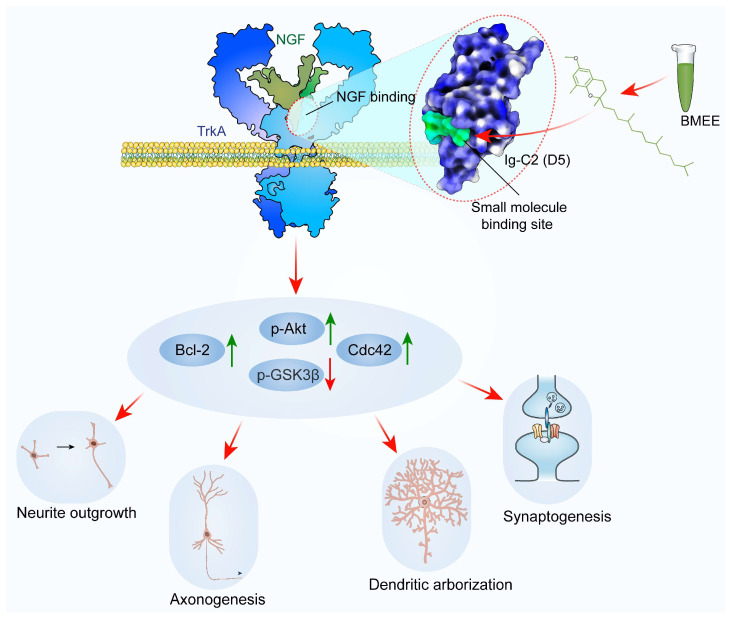
Mechanistic details of BMEE in neuronal development are illustrated in a schematic diagram. Using structural modeling and molecular dynamics (MD) simulations, prospective bioactive components interacting with the TrkA extracellular domain have been identified. Small molecules from BMEE, such as δ–Tocopherol, O–methyl–, can bind to and activate the NGF–binding site of the TrkA receptor. TrkA activation upregulates p–Akt, Bcl–2, and Cdc42 and downregulates p-GSK3β expression. Positive modulation of these neuronal signaling pathways promotes neurite outgrowth, axonogenesis, dendritic arborization, and synaptogenesis. Upward (green) and downward (red) arrows denote upregulation and downregulation, respectively.

Primary neuronal culture is an invaluable approach to investigating how neuronal morphology changes throughout development, aging, and degeneration [46], which undergo well-defined stages of morphological differentiation in vitro, with axon specification occurring around DIV3 and dendritic development becoming prominent by DIV5 [47,48]. Using this approach, the present study conducted a detailed investigation of *Bacopa monnieri* extract as a neurotrophic agent and revealed its mechanistic role in improving different important parameters of neurogenesis (Figure 10). Our results indicate that BMEE promotes neuronal growth in primary neurons, with the most evident effect observed at 2.5 μg/mL, without detectable cytotoxicity at the tested concentrations. This neurotrophic activity may partly underlie the traditional use of *Bacopa monnieri* in Ayurvedic medicine for brain-related disorders.

In traditional and Ayurvedic medicine, most treatments use natural ingredients for various diseases, while the detailed molecular mechanisms underlying these diseases remain to be explored. Recently, there has been growing evidence that network pharmacology is highly effective for studying the mechanisms of action of natural ingredients used in traditional and Ayurvedic medicine by focusing on a few critical pathways involved in disease perturbation [49,50,51]. This study, therefore, used network pharmacology approaches to gain mechanistic insight into how BMEE promotes neurogenesis, implicating cognitive function, learning ability, and memory [52,53]. To incorporate in silico network pharmacology, we first identified metabolites in BMEE using GC-MS, which revealed 22 compounds that interact with 743 targets identified via in silico target-fishing. By overlapping the compound target with targets of neuronal development, 93 shared targets were identified, which were associated with distinct signaling pathways involved in axonogenesis, dendrite formation, neuronal polarity, and maturation. Indeed, BMEE at 2.5 μg/mL significantly promotes axonogenesis and dendrite formation by increasing the number, length, and branching of axons and dendrites and by enhancing neuronal maturation during early differentiation. Although higher BMEE doses were used for the initial dose-optimization analysis, they were not considered for subsequent analyses due to neuronal death and toxicity.

Among the 93 neurodevelopment-related targets predicted by network pharmacology analysis, GSK3β, Bcl-2, Akt1, and Cdc42 were prioritized for experimental validation because they were highly connected nodes in the PPI network and were strongly associated with GO biological processes relevant to BMEE-mediated neuronal development, including neuritogenesis, axonogenesis, dendrite morphogenesis, and early neuronal differentiation. BMEE treatment indeed differentially regulated the expression of these four targets, including increased Bcl-2 and Cdc42 expression in BMEE-treated neurons, along with increased Akt1 phosphorylation. Cdc42 is involved in regulating several signals of neurogenesis, including axon formation [54], dendrite formation [55], spine development [56], and polarity [37]. Bcl-2, on the other hand, promotes cell survival during neuroprotection [57], and axonal outgrowth and regeneration [58]. It has been well documented that Akt1 is associated with neuroprotective signaling [39] and is a crucial mediator of neuronal polarity [59], branching, and elongation [60]. Although the overall Akt1 expression was not increased by BMEE treatment, BMEE increased Akt1 phosphorylation, suggesting that BMEE promotes Akt1 activation in a manner consistent with neurotrophin-mediated signaling [61,62]. On the other hand, BMEE treatment also decreased GSK3β phosphorylation, whose activity is critical for axonal outgrowth and neuronal branching [41]. It should be acknowledged that phosphorylation of Ser-9 of GSK3β decreases its functional activity [41,63], indicating that BMEE induced GSK3β activity. KEGG pathway analysis indicated that TrkA (*NTRK1*) may act as an upstream regulator of GSK3β, Bcl-2, Akt1, and Cdc42 within the neurotrophins signaling pathway. Consistent with this possibility, the BMEE-induced promotion of neuronal growth was abolished when neurons were co-treated with the TrkA-specific inhibitor GW441756. In addition, a molecular docking study followed by MD simulation suggested that BMEE metabolites, especially heptacosanoic acid, 25-methyl-, methyl ester; δ-Tocopherol, O-methyl-; and glycidyl palmitate, have a high affinity for the binding site for NGF-mimetic small molecule agonists at the fifth subdomain of TrkA receptor [64,65]. The compounds δ-Tocopherol, O-methyl-, and glycidyl palmitate maintained intermolecular interactions with several residues at the binding site, but only δ-Tocopherol, O-methyl- maintained interaction with a critical residue, which was previously reported to be important for the compound acting as an NGF mimetic [64,66,67]. This observation suggests that δ-Tocopherol O-methyl, which is abundantly present in BMEE, may be one of the bioactive components contributing to BMEE-mediated neuronal development and could potentially exert NGF-mimetic activity. Previous studies have shown that δ-Tocopherol O-methyl promotes neuronal differentiation and morphological maturation in cultured neurons [68,69]. However, direct evidence that δ-Tocopherol O-methyl interacts with or activates the TrkA receptor is currently lacking. Therefore, whether this compound functions as a true NGF mimetic remains to be determined in future studies.

Based on the above analysis, the present studies conclude that BMEE can significantly accelerate neuronal development activity by acting on TrkA-dependent pathways and promoting neuritogenesis, axonogenesis, and dendrite arborization (Figure 10). It has been reckoned that when axons and dendrites develop optimally, synaptic plasticity is enhanced, which improves memory functions [70,71]. Furthermore, TrkA activation also involves synaptic vesicle dynamics, neurotransmitter release, modulating receptor trafficking, synapse formation, and stability [72,73,74]. Similarly, we found that presynaptic/postsynaptic markers, SV2/PSD-95, as well as their co-localization at synapses, are upregulated in BMEE-treated neurons, and our further analysis revealed that BMEE positively upregulates both excitatory and inhibitory synapse formation. The number and density of both NMDA receptors (GluN2A and GluN2B) are increased significantly by BMEE. The number of inhibitory synapses (GAD6 and GABABR1) has also significantly increased. Previous studies reported that Bacopa can upregulate NMDA receptor expression in mouse brains [75] and play a role in synaptogenesis [17]. In Ayurvedic medicine, *Bacopa monnieri* has long been recognized for its memory-enhancing properties [76]. In agreement with previous studies, our findings support a beneficial role for BMEE in neuronal function and suggest that it may contribute to synaptic plasticity and long-term potentiation. Importantly, our data also raise the possibility that bioactive metabolites present in BMEE may stimulate multiple signaling pathways during neuritogenesis. However, the precise mechanism remains to be defined, as the present study does not distinguish whether BMEE enhances endogenous neurotrophin production, exerts NGF-mimetic effects through TrkA activation, or acts through additional parallel mechanisms. Accordingly, future studies will be required to identify the relevant secondary metabolites of *Bacopa monnieri*, directly assess endogenous neurotrophin levels, and clarify the involvement of other signaling pathways in BMEE-mediated neuronal development.

## 4. Materials and Methods

### 4.1. Collection of Plant Materials and Extract Preparation

Whole plants of *Bacopa monnieri* were collected in Dhaka in January 2012 and identified by experts at the Bangladesh National Herbarium. The plant name was verified at www.plantlist.org, accessed on 13 April 2021. After collection, the plants were carefully cleaned, air-dried in a shady area, and ground in a grinder. To prepare the extract, 3000 mg of powdered plant material was soaked in 70% ethanol at a solvent-to-material ratio of 1:10 (*w*/*v*) and shaken at 200 RPM at room temperature. The extraction process was continued for 24 h. These extracts were filtered with filter paper and dried at room temperature. The prepared dried extract was stored at −20 °C in an airtight tube. The voucher specimen was placed in the Department of Anatomy (Il-Soo Moon’s Lab, Gyeongju, Republic of Korea). To prepare a working solution of the dried extract at 8 mg/mL, the stock solution was further diluted to obtain other working solutions in DMSO.

For in vitro experiments, the dried extract was first dissolved in 70% ethanol to prepare a primary stock solution (8 mg/mL), which was subsequently diluted in DMSO to generate working stock solutions. These working stocks were added to the culture medium to obtain final BMEE concentrations of 0.2, 0.5, 1, and 2.5 μg/mL. The final concentration of DMSO in the culture medium was maintained at 0.5% (*v*/*v*) for all treatment and vehicle control groups, whereas the residual ethanol concentration was negligible. Vehicle control cultures received the same final concentration of DMSO without BMEE.

### 4.2. Primary Culture of Neurons and Extract Preparation

Study approval was granted by the Institutional Animal Care and Use Committee, College of Medicine, Dongguk University (acceptance certificate number DUK-IBC-21-001). Pregnant Sprague-Dawley rats (E-13) were received from the supplier and housed individually at controlled temperatures on a 12/12 h light–dark cycle, with free access to food and water at Dongguk Medical College (Gyeongju, Republic of Korea). On the 19th day of gestation (E-19), the rat was euthanized with isoflurane and sacrificed by cervical dislocation. The embryos were collected in ice-cold Hank’s balanced salt solution (HBSS), and primary hippocampal neurons were subsequently isolated from them. Neurons were obtained from three independent litters, with embryos from each litter pooled; each litter was considered one biological replicate (*n* = 3). Embryonic sex was not determined, and cultures therefore contained mixed-sex embryos. The embryos were carefully moved to a sterile laminar hood, their heads were cut off with scissors, and the cortex and hippocampus were carefully dissected under a Leica stereo microscope at room temperature to ensure precise neuron extraction. Collected hippocampi were cut into small pieces using sterile sharp forceps, digested by incubation in 0.25% trypsin for 30 min, and triturated using fire-polished Pasteur pipettes.

Culture media were pre-plated with indicated doses of BMEE or vehicles (DMSO, 0.5%). We used 12 mm coverslips coated with poly-D-lysine (PDL) to seed cells for immunocytochemistry, morphology, and viability studies with a seeding density of 3.0 × 10^4^ cells/cm^2^ in 800 µL of a defined medium in 24-well plates [77,78]. For the Western blot, 3.0 × 10^6^ cells/cm^2^ were seeded onto PDL-coated six-well culture plates. Cultures were incubated at 37 °C for the indicated days under atmospheric conditions of 5% CO_2_ and 95% air in a serum-free Neurobasal medium supplemented with B27. Culture media and specified concentrations of BMEE were replaced every 3 days throughout the culture period.

### 4.3. Neuronal Viability by Trypan Blue

A trypan blue exclusion assay was used to determine neuronal viability at in vitro day 8 (DIV 8). Cells were stained with 0.4% trypan blue for 15 min at RT. Phase-contrast microscopy was used to acquire pictures after the cultures were rinsed with Dulbecco’s phosphate-buffered saline (D-PBS, Invitrogen, Eugene, OR, USA). From the images, the number of viable and dead cells was counted. The proportion of viable cells was determined by dividing the total number of cells enumerated (including both live and dead neurons) by the number of unstained cells (living neurons). In each experiment, three coverslips were used to randomly count 500 cells.

### 4.4. Characterization of the Extract by Gas Chromatography and Mass Spectrometry (GC-MS)

GC-MS assessed the active constituents of BMEE. The GC-MS machinery used in this study consists of a 7890A capillary gas chromatographic system (Agilent Technologies, located in Santa Clara, CA, USA), a mass spectrometer consisting of 95% dimethyl-poly-siloxane and 5% phenyl, and a fitted silica capillary column (0.25 mm diameter and 90 m length, thin film thickness of HP-5MSI 0.25 µm). The system used helium gas of 99.99% purity at a flow rate of 1 mL/min. The injection port temperature was 250 °C.

The iron-source temperature was 280 °C. The column temperature was held at 110 °C for 2 min and then increased to 200 °C at 10 °C/min. Afterwards, the final temperature reached 280 °C, followed by a slower rate of 5 °C/min, which was kept for 9 min. The MS range was 50–550 *m*/*z*, and the elution time was 45 min. To identify the compounds present, the mass spectra of the unidentified peaks were compared to the extensive databases maintained by the National Institute of Standards and Technology (NIST).

### 4.5. In Silico Target Fishing and Network Building

The compounds identified from GC-MS have been subjected to three popular in silico target-predicting servers, TargetNet [79] (http://targetnet.scbdd.com/, accessed on 11 August 2022), SwissTargetPrediction [80] (http://www.swisstargetprediction.ch/, accessed on 11 August 2022), and SEA [81] (https://sea.bkslab.org/, accessed on 11 August 2022), to predict the potential targets. TargetNet analyzed the QSAR models to identify a possible composite target using chemogenomic data as input. SwissTargetPrediction predicts the likely macromolecular targets from 2D and 3D similarity alignments when comparing small molecules to a library of bioactive compounds. SEA uses a chemical similarity approach to predict chemical targets. For target prediction, a cutoff value of ≥0.5 was applied for TargetNet. In SwissTargetPrediction, targets with a probability score ≥ 0.10 were selected, while in SEA, targets with an E-value ≤ 10^−3^ were considered significant. A compound–target network was built using Cytoscape 3.7.0. After that, the compound–target–neuronal development signaling pathways (C-T-ND) network was constructed by merging a curated neuronal development network from the gene ontology database with the compound–target network [82,83]. To build an active C-T-ND network, we used Cytoscape v3.7.0 and the STRING database to identify targets associated with neuronal development that overlap with compound targets, with a confidence score of 0.4. The topological properties of the constructed network were evaluated using the NetworkAnalyzer tool of Cytoscape 3.7.0. The C-T-ND network and the cluster analysis-identified targets were placed in the Visualization and Integrated Discovery DAVID 6.8 [84] (https://davidbioinformatics.nih.gov/, accessed on 11 August 2022) database for GO enrichment and KEGG (Kyoto Encyclopedia of Genes and Genomes; https://www.kegg.jp/ or https://www.genome.jp/kegg/, accessed on 11 August 2022) [85] pathway enrichment analyses. The enrichment analysis results of this study were screened at *p <* 0.05 and specific pathways [86].

### 4.6. Immunocytochemistry

Hippocampal neurons in culture were fixed at DIV3/5/14 using a combination of 4% paraformaldehyde and methanol, followed by blocking with 2.4% goat serum, as previously detailed, for immunostaining [87]. The following antibodies were used for immunostaining: primary antibodies to Ankyrine G (rabbit polyclonal H-215, 1:50 dilution; Santa Cruz Biotechnology Incz., Santa Cruz, CA, USA); tubulin α-subunit (mouse monoclonal 12G10, 1:20 dilution; Developmental Studies Hybridoma Bank, University of Iowa, USA); Synaptic vesicle 2 (mouse monoclonal SV2, 1:100; Developmental Studies Hybridoma Bank, University of Iowa, Iowa City, IA, USA); Postsynaptic density-95 (rabbit polyclonal PSD-95; 1:1000; Upstate Biotechnology Inc., Lake Placid, NY, USA); Anti-Map2 (Chicken Monoclonal Map2, 1:2000, Abcam, Cambridge, UK); GluN2A and GluN2B (rabbit polyclonal, 1:500 [88,89]); GAD6 (mouse polyclonal, 1:500, Developmental Studies Hybridoma Bank, University of Iowa, USA); GABAB receptor 1 (GABABR1, rabbit polyclonal, 1:100; Chemicon, Millipore, MA, USA); p-GSK3β (Ser9) (rabbit polyclonal, 1:500, Cell signaling, Danvers, MA, USA); Bcl-2 (rabbit polyclonal, 1:100, Abclonal, Woburn, MA, USA); Cdc42 (rabbit polyclonal, 1:50, Thermofisher, Waltham, MA, USA); Akt (rabbit polyclonal, 1:100, Cell signaling, MA, USA); p-Akt (Ser473) (rabbit polyclonal, 1:100, Cell signaling, MA, USA); and secondary antibodies (Alexa Fluor 488-conjugated goat anti-mouse IgG [1:1000] and Alexa Fluor 568-conjugated donkey anti-rabbit IgG [1:1000], Molecular Probes, Eugene, OR, USA). Following overnight incubation with primary antibodies, neurons were incubated for 2 h with secondary antibodies before being mounted on slides.

### 4.7. Fluorescence Labeling of Neurons with Dil

At DIV3, neurons were live-stained with Vybrant Dil (Molecular Probes, Invitrogen, Eugene, OR, USA) per the manufacturer’s instructions. When it attaches to cell plasma membranes, the lipophilic dye Dil (Benzoxazolium,3-octadecyl-2-[3-(3-octadecyl-2(3H)-benzoxazolylidene)-1-propenyl]-, perchlorate/34215 57-1) can be observed, allowing for the imaging of whole neurons.

### 4.8. Protein Extraction and Immunoblotting

The cells were grown until DIV 16 in culture medium containing BMEE. After the indicated time, cells were collected by scrappers in RIPA buffer [50 mM Tris-HCl (pH 8.0), 150 mM NaCl, 1% (*v*/*v*) NP-40, 0.5% (*w*/*v*) sodium deoxycholate and 1% (*w*/*v*) sodium dodecyl sulfate (all from Thermo Scientific, Rockford, IL, USA)] and kept for 15 min on ice, followed by (10 min at 4 °C at 12,000 rpm) centrifugation. The protein concentration was calculated using the Bradford method after centrifugation at 12,000 rpm and 4 °C for 10 min [90]. SDS-Polyacrylamide gels were loaded after dissolving 30 micrograms of total protein in sample buffer and then boiling for 5 min. The proteins were transferred from the SDS-PAGE to the PVDF membranes after protein loading. The membranes were then blocked with 5% non-fat dry milk in Tris-buffered saline/0.05% Tween 20 (TBST). The membrane was probed with indicated antibodies (1:1000), followed by the addition of horseradish peroxidase (HRP) secondary antibodies, followed by chemiluminescence (ECL) detection with the West-Q Pico ECL Solution from Gendepot (Gendepot, Katy, TX, USA).

### 4.9. Image Acquisition and Analysis

An image-based analysis was performed using Image J (1.45), a simple neurite tracer plug-in (National Institute of Health, Bethesda, MD, USA) was used, and the Sholl plug-in was used (http://ghoshlab.org/software/, accessed on 11 August 2022). An examination of morphological parameters such as primary dendrite length (sum of the length of the primary neurites) and axonal length revealed that primary dendrites provide the bulk of the neurites formed in neurons. Axonal and dendritic branching orders were also analyzed. Digital images of stained samples were obtained using a Leica Research Microscope DM IRE2 (Leica Microsystems AG, Wetzlar, Germany) and a high-resolution CoolSNAP^TM^ CCD camera (Photometrics Inc., Tucson, AZ, USA) and LASX software (version 3.8.1.0, Leica, Wetzlar, Germany).

Thirty neurons from different neuronal cultures’ phase-contrast microscopic images were selected for morphometric analysis, and fifteen for Sholl analysis and statistical analysis. From the statistical analysis, the 2.5 µg/mL concentration showed the most significant effect across all parameters and was therefore chosen for further analysis. For synapse analysis, colocalized pre- and postsynaptic puncta were counted by selecting a 50 µm long segment from three regions in 10 different neurons. The neurons were stained with Dil to observe morphology, as shown in Figure 1B.

### 4.10. Molecular Docking and Molecular Dynamics (MD) Simulation

The Glide XP docking technique was used for molecular analysis using Schrödinger Suite, Ver 2022-4 (Schrödinger, LLC, New York, NY, USA). Metabolite structures were prepared for docking using Ligprep tools after being acquired in 3D format from PubChem. The crystal structure of the fifth domain of the TrkA receptor was retrieved from the Protein Data Bank (PDB id: 1WWW) and prepared for docking calculations by using protein preparation tools of Schrödinger Suite and subjected to Glide XP docking following the procedure described earlier [13]. Briefly, the structure was preprocessed by adding appropriate hydrogen atoms, assigning charges, and correcting bond orders, while water molecules located more than 5 Å from the active site were removed. The structure was then optimized using PROPKA at neutral pH. Final restrained minimization was performed with the OPLS 4 force field, with heavy atoms constrained to converge to an RMSD of 0.30 Å. For docking, the protein active site was defined by generating a grid box centered on the binding site of the reference ligand [67]. Grid generation after minimization was carried out using the default parameters, with a box size of 15 Å × 15 Å × 15 Å, and the OPLS 4 force field was applied during this step. The charge cutoff and van der Waals scaling factor were set to 0.25 and 1.00, respectively.

Molecular dynamics (MD) simulation was conducted utilizing the OPLS4 force field in Desmond software version 2023-4 [91]. We used the transferable intermolecular potential 3-point (TIP3P) water model to solvate the protein–ligand complex structure in an orthorhombic box with a 10 Å extension on either side [92]. The solvation system was kept in NPT condition, with the pressure set at 1 atm and the temperature at 300 K. This was achieved using the Nose–Hoover thermostat [93] and Martyna–Tobias–Klein barostat [94]. The system was adjusted to physiological conditions by adding Na^+^ and Cl^−^ ions to 0.15 M. The system underwent minimization and equilibration using the standard serial minimization protocol of Desmond [95]. For determining the long-range electrostatic interactions, the particle mesh Ewald method [96] was used. As for the short-range electrostatic interactions, a cutoff of 9.0 Å was employed. For integrating the equations of motion, the multistep RESPA integrator [97] was used. It handled both bonded and non-bonded interactions within the short-range cutoff. The integration was carried out with an inner time step of 2.0 fs. Finally, a 300 ns molecular dynamics simulation was performed using the NPT ensemble with three independent repeats. The last frame of each run was used as the starting structure for the subsequent run, and trajectories were recorded every 100 ps. System stability was evaluated by calculating the root mean square deviation (RMSD) of the Cα atoms relative to the starting structure throughout the simulation trajectory. Protein–ligand interactions were analyzed using the Simulation Interaction Diagram module of the Schrödinger Suite. Both the docked complex and the last 100 ns of the simulated trajectories (1000 frames) were subjected to MM-GBSA analysis to calculate binding energies, following a similar approach described earlier [13].

### 4.11. Statistical Analysis

All experiments were performed three times, with three replicates per experiment, and the data are presented as the mean ± SEM across the three independent experiments. Statistical analyses were performed using GraphPad Prism version 8.0.2 for Windows. Comparisons between two groups were analyzed using an unpaired two-tailed Student’s *t*-test. Comparisons among more than two groups were performed using one-way ANOVA followed by the appropriate post hoc multiple-comparison test.

## 5. Conclusions

The present study uses a combined network pharmacology and in vitro primary neuronal culture approach to explain how BMEE improves memory function and ameliorates cognitive deficits. Our study identifies that BMEE accelerates neurite outgrowth, promotes axonogenesis and dendritic arborization, and regulates synaptic architecture. We also demonstrated that the activation of the TrkA receptor and subsequent modulation of downstream signaling pathways, including Bcl-2, Cdc42, Akt1, and phosphorylation of GSK3β and Akt1, were the primary mechanisms by which BMEE-mediated neuritogenic activity was exerted. This was achieved by combining network pharmacology with in vitro studies. Molecular modeling and structural dynamics studies provide evidence that BMEE secondary metabolites, especially δ-Tocopherol O-methyl-, have a high affinity for the TrkA receptor and could act as potential neurotropic factor mimetic agents. Indeed, more research on BMEE is needed to establish effective treatments for many types of cognitive diseases, but our studies suggest that Bacopa monnieri could be a potential source of new drugs that mimic neurotrophic factors.

## Data Availability

The original contributions presented in this study are included in the article/Supplementary Material. Further inquiries can be directed to the corresponding author.

## Supplementary Materials

The following supporting information can be downloaded at https://www.mdpi.com/article/10.3390/ijms27073048/s1.
