# Supplementary material for: Bacopa monnieri Promotes Neuronal Development by Regulating the Neurotrophin Signaling Pathway
Source: Int J Mol Sci. 2026 Mar 27;27(7):3048. doi: 10.3390/ijms27073048 (PMC13072882; doi:10.3390/ijms27073048)
Supplement: Supplementary file 1 [file ijms-27-03048-s001.zip › Supplementary File S2.pdf]

Supplementary File S2

Table S1: List of compounds identified from GC-MS analysis along with their retention time, peak area, and 2D structure

Serial No.	Compound Name	RT(min)	Peak Area(%)	2D Structure
1	BENZENE, 1,3-DIMETHYL-	4.28	2.846479	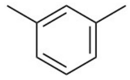
2	BENZENE, 1,3-DIMETHYL-	4.85	0.60673	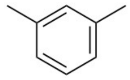
3	HEPTANE, 2,2,4-TRIMETHYL-	5.13	0.136981	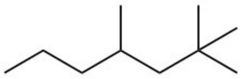
4	HEPTANE, 4-ETHYL-2,2,6,6-TETRAMETHYL-	7.2	0.311385	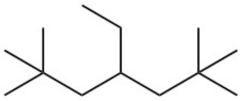
5	HEXANE, 1,1-DIETHOXY-	8.18	0.464156	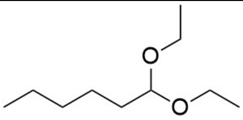
6	HENTRIACONTANE	9.7	1.781281	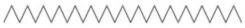
7	DIETHYLCYANAMIDE	9.8		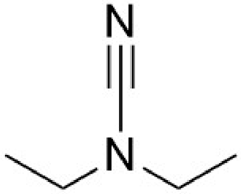
8	LINALOOL	9.17	3.628965	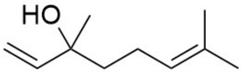
9	CYCLOPENTANECARBOXYLIC ACID, 2-TETRAHYDROFURYLMETHYL ESTER	9.33		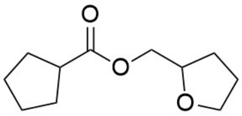
10	BUTYL PENTADECANOATE	9.59		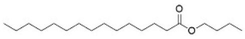

11	CYCLOPENTANE, 1-ETHYL-1-METHYL-	14.9	0.183759	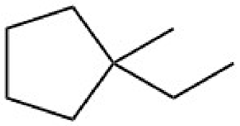
12	DICHLORINE HEPTOXIDE	14.66		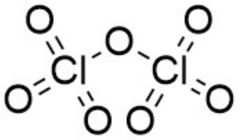
13	HENTRIACONTANE	14.78	0.398753	
14	PROPANE, 2-CHLORO-2-NITRO-	19.6		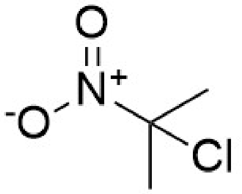
15	PROPANE, 2-CHLORO-2-NITRO-	19.55	0.307057	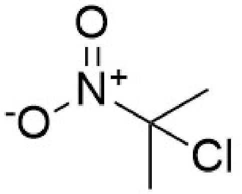
16	OCTADECANOIC ACID, 2-OXO-, METHYL ESTER	20.5		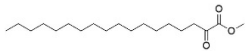
17	2,4-DI-TERT-BUTYLPHENOL	20.6	0.466846	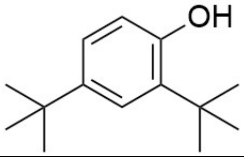
18	2,4-DI-TERT-BUTYLPHENOL	25.02	0.096645	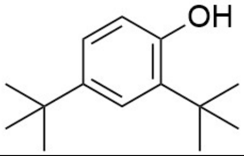
19	3-METHYL-2-(2-OXOPROPYL)FURAN	25.15	0.29585	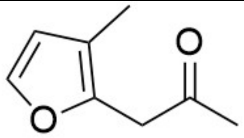
20	1,5-DIPHENYL-2H-1,2,4-TRIAZOLINE-3-THIONE	26.4	0.119823	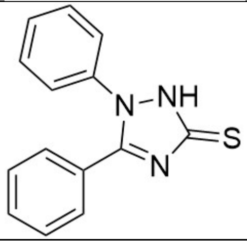

21	1,9-NONANEDIOL, DIMETHANESULFONATE	26.13	0.088549	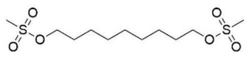
22	4-METHOXY-6-METHYL-6,7-DIHYDRO-4H-FURO[3,2-C]PYRAN	26.27	0.082343	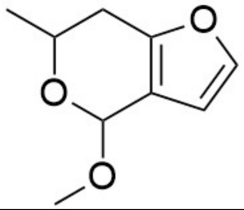
23	HEPTACOSANOIC ACID, 25-METHYL-, METHYL ESTER	27.9	0.288986	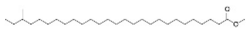
24	ETHYL 15-METHYL-HEXADECANOATE	29.1	2.167865	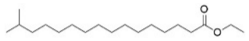
25	3-METHYL-2-(2-OXOPROPYL)FURAN	32.7		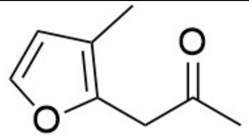
26	8-BROMOOCTANOIC ACID, ETHYL ESTER	32.615	2.311803	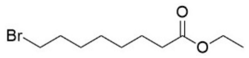
27	9-OCTADECENAMIDE, (Z)-	34.9	13.65366	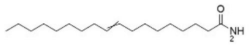
28	GLYCIDYL PALMITATE	35.55	0.898837	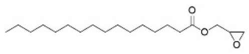
29	.DELTA.-TOCOPHEROL, O-METHYL-	37.8	12.39302	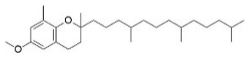

Note: Compounds listed with two retention times, might be attributable to the presence of isomeric or closely related compounds producing similar mass spectra.

Table S2: List of compounds after removing duplicates and their short name used in the network pharmacology

Name of Compound	Short Name in Network Pharmacology
δ-Tocopherol, O-methyl-	C1
1,5-Diphenyl-2H-1,2,4-triazoline-3-thione	C2
2,4-Di-tert-butylphenol	C3
3-Methyl-2-(2-oxopropyl)furan	C4
4-Methoxy-6-methyl-6,7-dihydro-4H-furo[3,2-c]pyran	C5
9-Octadecenamide, (Z)-	C6
Benzene, 1,3-dimethyl-	C7
Butyl pentadecanoate	C8
Cyclopentane, 1-ethyl-1-methyl-	C9
Cyclopentanecarboxylic acid, 2-tetrahydrofurylmethyl ester	C10
Dichlorine heptoxide	C11
Diethylcyanamide	C12
Ethyl 15-methylhexadecanoate	C13
Glycidyl palmitate	C14
Hentriacontane	C15
Heptacosanoic acid, 25-methyl-, methyl ester	C16
Heptane, 2,2,4-trimethyl-	C17
Heptane, 4-ethyl-2,2,6,6-tetramethyl-	C18
Hexane, 1,1-diethoxy-	C19
Linalool	C20
Octadecanoic acid, 2-oxo-, methyl ester	C21
Propane, 2-chloro-2-nitro-	C22
1,9-Nonanediol, dimethanesulfonate	C23
8-Bromooctanoic acid, ethyl ester	C24

Table S3: Result of MM-GBSA binding energy calculation of the compounds after molecular docking.

Compound Name	MM-GBSA (kcal mol^{-1})
9-Octadecenamide	-36.22
Linalool	-22.96
2,4-Di-tert-butylphenol	-27.29
Benzene, 1,3-dimethyl-	-20.1
2-Chloro-2-nitropropane	-14.29
Diethylcyanamide	-17.9
Hentriacontane	-46.75
Nonasulphan	-28.43
2,2,4-Trimethylheptane	-17.95
1-Ethyl-1-methylcyclopentane	-15.73
4-Ethyl-2,2,6,6-tetramethylheptane	-18.08
1,1-Diethoxyhexane	-23.96
Chlorine heptoxide	-15.75
Ethyl 8-bromooctanoate	-26.32
Glycidyl palmitate	-37.73
3-Methyl-2-(2-oxopropyl)furan	-21.64
Cyclopentanecarboxylic acid, 2-tetrahydrofurylmethyl ester	-29.93
19-Norpregna-1,3,5(10)-trien-20-yne-3,17-diol, (17alpha)	-39.65
Methyl 2-oxooctadecanoate	-32.07
2,3-Diphenyl-1H-1,2,4-triazole-5-thione	-24.24
Butyl pentadecanoate	-27.28
Ethyl 15-methyl-hexadecanoate	-31.23
δ-Tocopherol, O-methyl-	-46.6

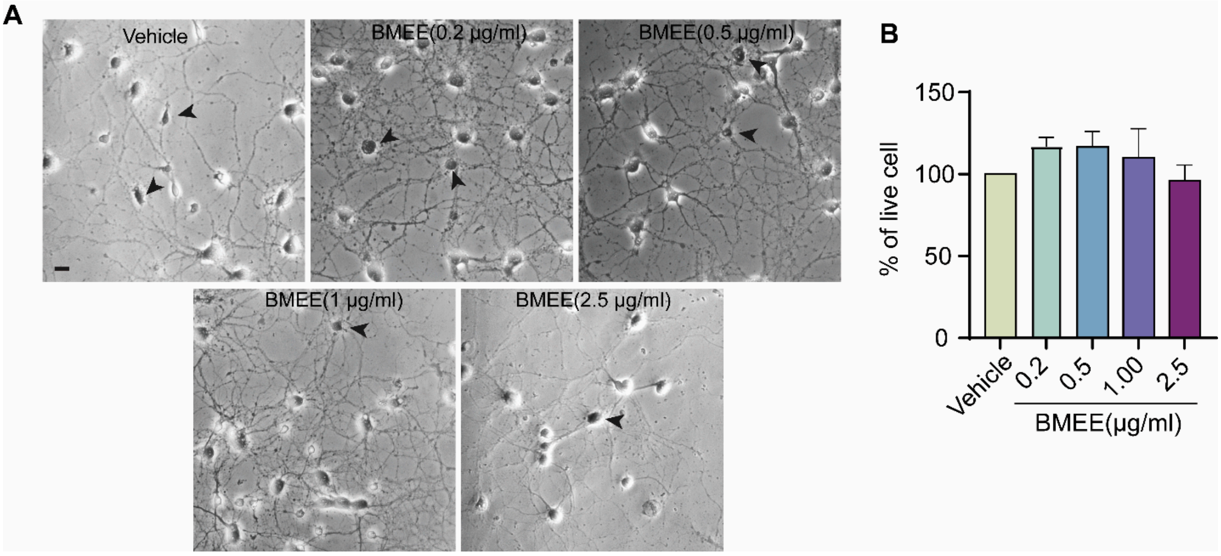

Figure S1: (A) Phase-contrast images of primary cultured neurons grown until DIV 8, and stained with trypan blue. Cells were treated with different doses of BMEE (0, 0.2, 0.5, 1, 2.5 µg/ml). (B) The bar diagram shows the viabilities of each treatment condition presented as percentages and were calculated by expressing the number of unstained cells as a percentage of the total number of cells (live±dead neurons). Each experiment randomly counted 500 cells per coverslip on three coverslips.

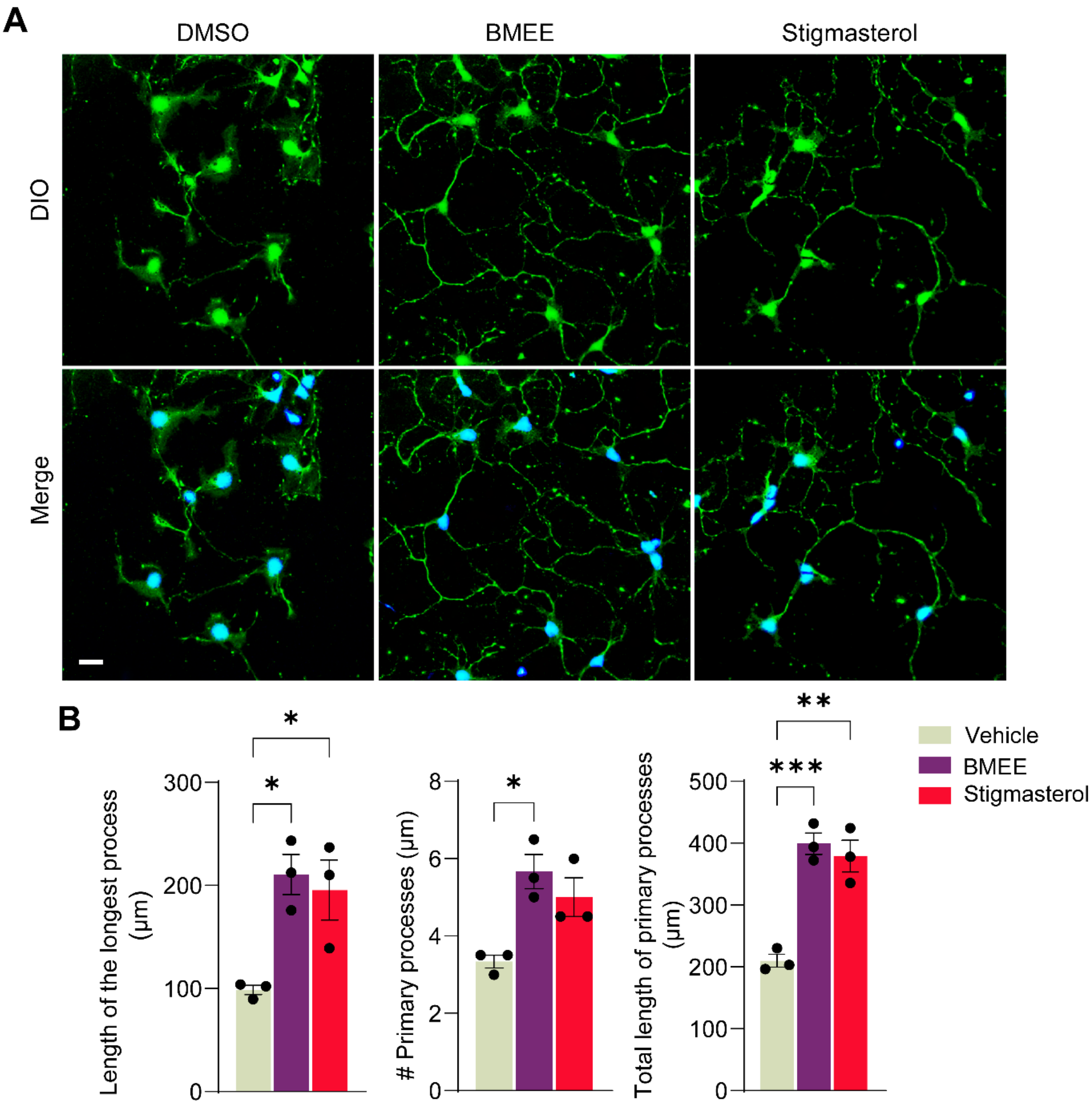

Figure S2. (A) Fluorescent images of primary cultured neurons of DIV3, Fixed and stained with Dio (green) and DAPI (blue). Cells were treated with BMEE (2.5 μg/ml), Stigmasterol (50 μm), and vehicle. Scale bar=20 μm. Stigmasterol is used as a positive control. (B) Statistical analysis of neuron parameters, such as the length of the longest process, the number of primary processes, and the total length of all the processes. The significance of the statistical test was obtained by using a two-tailed Student's t-test (*$p < .05$, **$p < .01$, ***$p < .001$). Each experiment was performed thrice (n = 3, each including 10 neurons). Bar represents the mean ± SEM (standard error of the mean) from three independent experiments. “#” denotes number of.

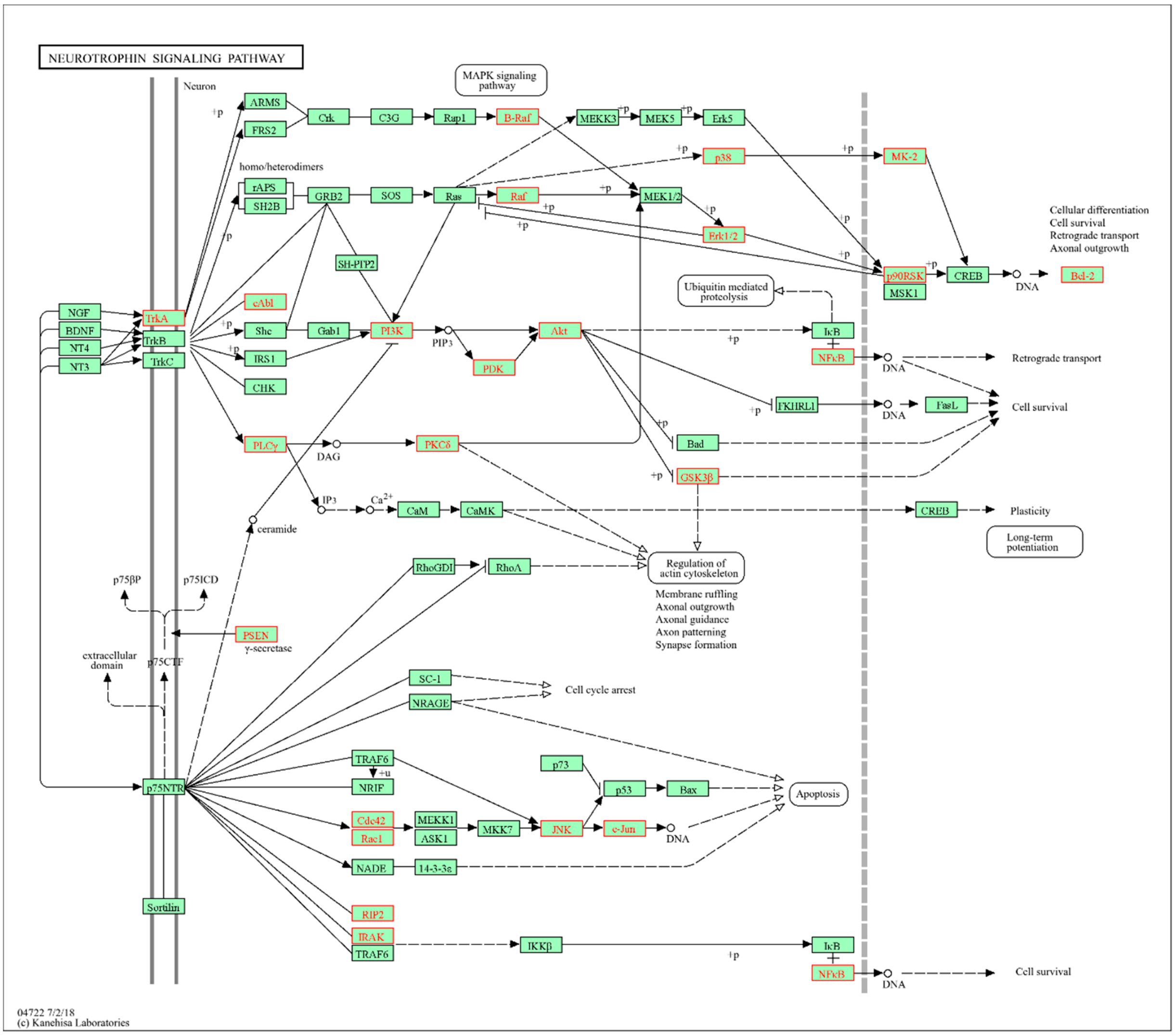

Figure S3. KEGG pathway mapper showing the involvement of BMEE targets in the Neurotrophin signaling pathway. BMEE targets are highlighted in the Red box.

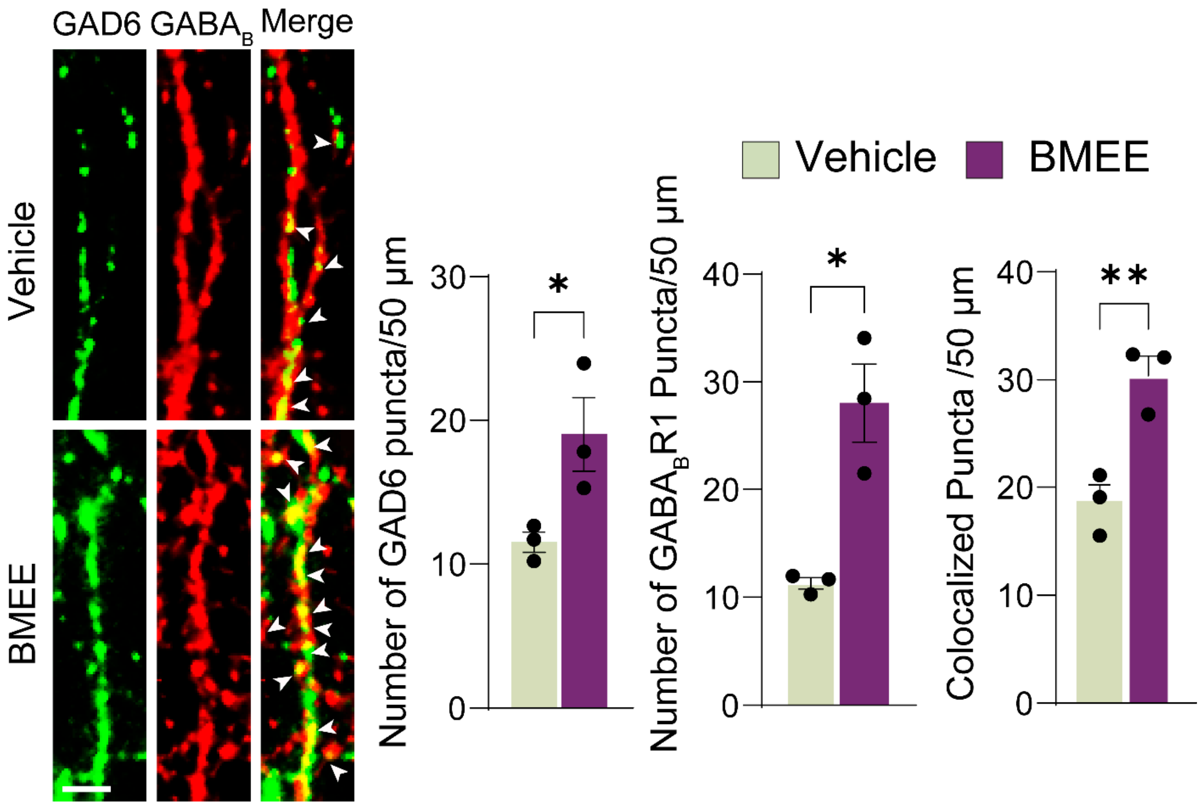

Figure S4. BMEE improves the expression of GABAergic synapse formation. Primary cultured neurons grown until DIV16, treated with BMEE or vehicle Fixed and immunostained with pre-synaptic and postsynaptic markers, GAD6 (green) and GABA$_B$R1 (red), respectively. The co-localized puncta represent a synapse marked by a white arrow. Statistical analysis represents puncta numbers along 50 μm dendrite length for GAD6, GABA$_B$R1, and synapse. The significance of the statistical test was obtained by using a two-tailed Student's t-test ($p < .05$, $p < .01$, $p < .001$). Each experiment was performed thrice (n = 3, each including 10 neurons). Bar represents the mean ± SEM (standard error of the mean) from three independent experiments. Scale bar, 2 μm, applies to all images.

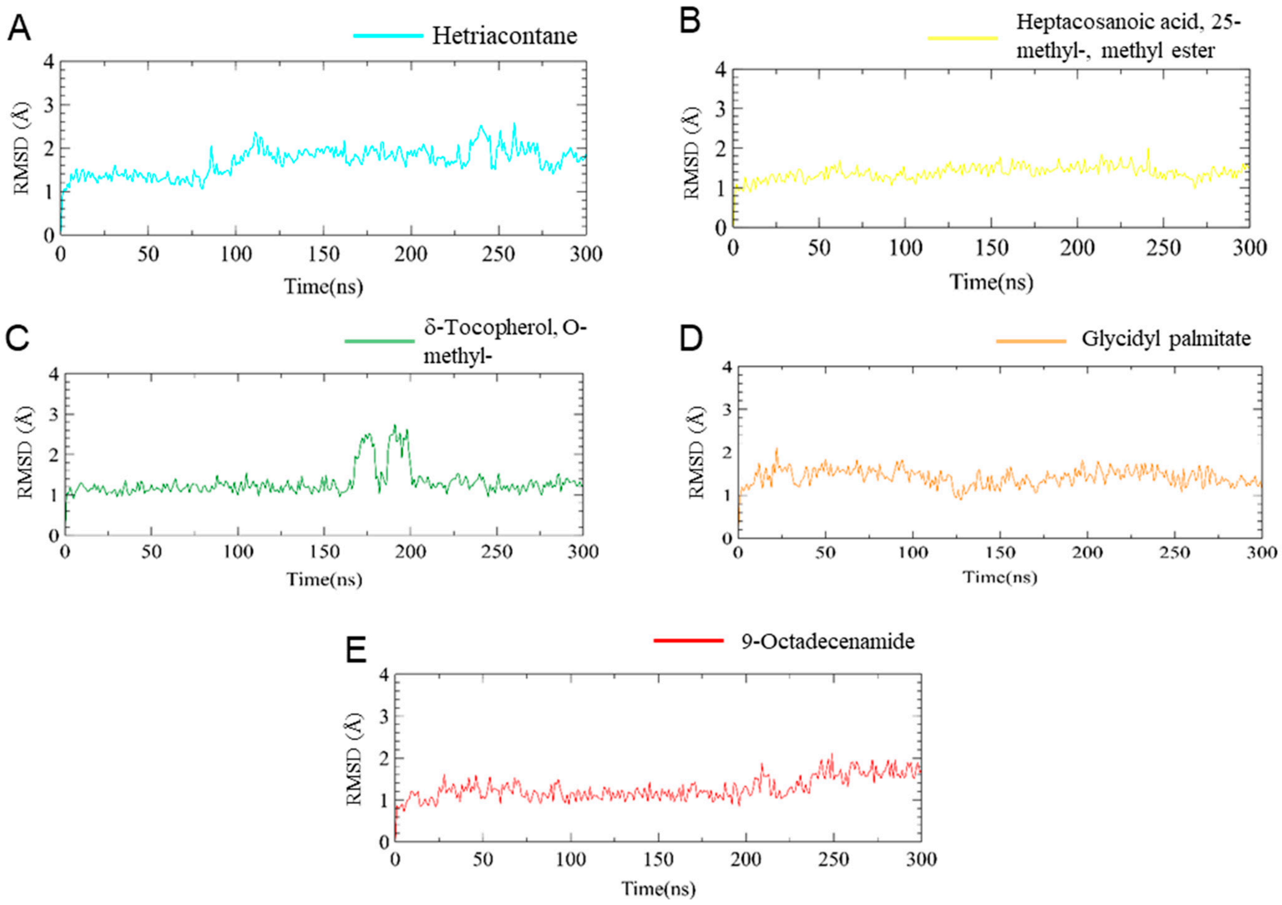

Figure S5. The RMSD values from the simulation of the top five hits, including (A) Hentriacontane, (B) Heptacosanoic acid, 25-methyl-, methyl ester, (C) δ-Tocopherol, O-methyl- (D) Glycidyl palmitate, (E) 9-Octadecenamide, (z)-, were calculated from each run of the specific system utilizing the protein c-alpha, respectively.

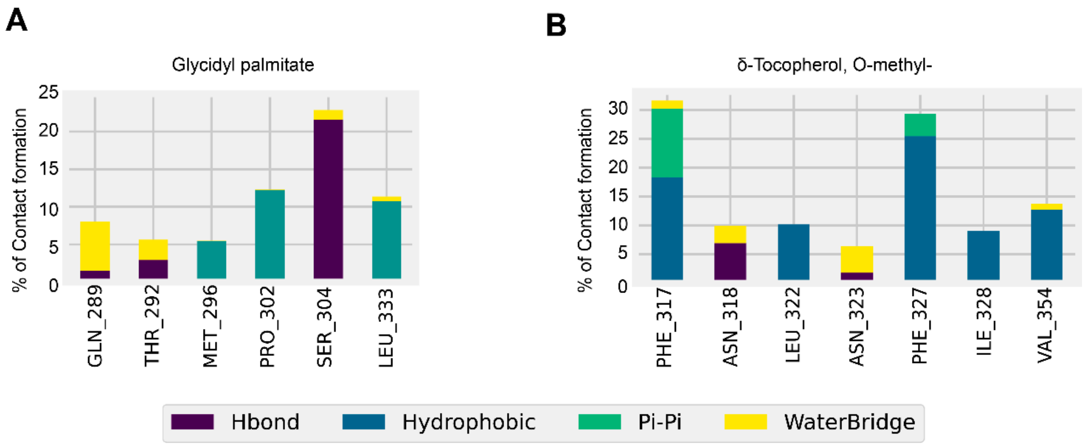

Figure S6. Percentage of total contact formation during the simulation for (A) Glycidyl palmitate, (B) δ-Tocopherol, O-methyl-, respectively.
